# Development and Validation of an HPLC-MS/MS Method for the Simultaneous Quantification of Vitexin and Isovitexin in Rabbit Plasma: Pharmacokinetic Insights on a Microcapsule Formulation

**DOI:** 10.3390/molecules30081690

**Published:** 2025-04-10

**Authors:** Duc Tuan Nguyen, Trung Nguyen Nguyen Le, Duc Kien Ngo, Hien Minh Khuu, Khang Thien Tran, Hoang Thanh Le, Hung Viet Tran, Truong-Thang Nguyen Phan, Vo Thi Kim Khuyen, Han Hoang Do, Nhu Huynh Mai, Quan Minh Le

**Affiliations:** 1 Faculty of Pharmacy, University of Medicine and Pharmacy at Ho Chi Minh City, Ho Chi Minh City 700000, Vietnam; ductuan@ump.edu.vn (D.T.N.); ngokienduc@ump.edu.vn (D.K.N.); kmhien.d20@ump.edu.vn (H.M.K.); vtkkhuyen@ump.edu.vn (V.T.K.K.); dhhan@ump.edu.vn (H.H.D.); mhnhu@ump.edu.vn (N.H.M.); 2 Institute of Drug Quality Control, Ho Chi Minh City 700000, Vietnam; trungnguyenlenguyen@gmail.com (T.N.N.L.); ttkhangfw@gmail.com (K.T.T.); lthoang1407@gmail.com (H.T.L.); tran.viethung168@gmail.com (H.V.T.); pntruongthang@gmail.com (T.-T.N.P.)

**Keywords:** vitexin, isovitexin, LC-MS/MS, pharmacokinetic, encapsulation, rabbit plasma

## Abstract

Vitexin and isovitexin are natural flavone C-glucosides that have numerous benefits for human health. However, their low oral bioavailability and poor gastrointestinal absorption dramatically restrict their potential medicinal uses. To overcome this challenge, chitosan-coated alginate microcapsules were prepared for intragastrical administration to rabbits. An LC-MS/MS method was developed and validated for the simultaneous determination of vitexin and isovitexin in the plasma of treated rabbits, using salicylic acid as the internal standard. Raw rabbit plasma samples were deproteinized using acetonitrile as a precipitation agent. Chromatographic separation was performed on a reversed-phase C18 column (100 mm × 4.6 mm, 3.5 µm), with an isocratic mobile solvent system comprising methanol and 0.1% acetic acid (40:60) as the mobile phase. All the analytes and the internal standard were ionized on a triple quadrupole mass spectrometer and electrospray ionization, operating in negative mode and multiple reaction monitoring. The analytical approach developed underwent validation in terms of system suitability, specificity, selectivity, LLOQ of 2 ng/mL, linearity (2.0–200 ng/mL, R^2^ > 0.99), accuracy (the intra- and inter-day from 94 to 110% with the relative standard deviations no more than 8.7%, precision with the recoveries from 97% to 102%, matrix effect (90–100%), carry-over, dilution integrity (2 times), and stability at room and frozen temperature for up to 1 month, and all the parameters met FDA and EMA requirements for bioanalytical methods. The validated procedure was applied to measure the absorption of vitexin and isovitexin from encapsulated extracts in a pilot pharmacokinetic study on rabbit plasma. Compared to the raw traditional extracts, the microcapsules enhanced the bioavailability of vi-texin and isovitexin regarding C_max_ and AUC values.

## 1. Introduction

**Figure 1:**
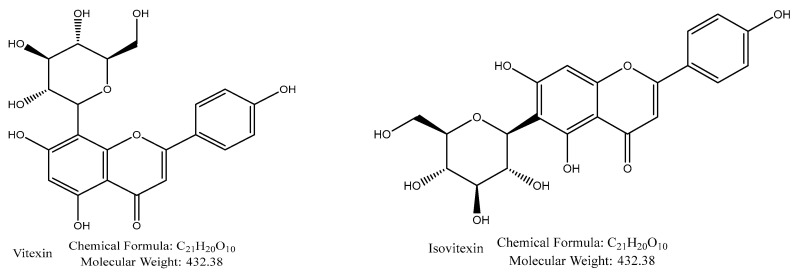
Chemical structure of vitexin and isovitexin.

Vitexin (8-C-glucosyl apigenin) and isovitexin (6-C-glucosyl apigenin) (Figure 1) are naturally occurring C-glycosyl flavones with numerous benefits for human health that have been extracted from various herbal plants such as *Xanthosoma violaceum* [1], pigeonpea [2], and *Santalum album* L. leaves [3,4]. These compounds exhibit diverse pharmacological properties, including anti-inflammatory [3], antioxidant [2,5], antimicrobial, radioprotective, and anticancer activities [2,6]. Notably, they are promising natural agents for managing diabetes mellitus due to the multitargeted effects demonstrated in both in vitro and in vivo studies [7]. However, the poor absorption of vitexin and isovitexin in the gastrointestinal tract and their low oral bioavailability limit their therapeutic application [7]. To address these limitations, various strategies to enhance the bioavailability of vitexin and isovitexin, such as nanolization [8], encapsulation [9,10], and β-cyclodextrin inclusion [11,12], have been explored. These approaches have shown promise in improving the water solubility of both vitexin and isovitexin in in vitro studies; however, in in vivo conditions, their efficacy in advanced drug delivery systems is scarce, posing challenges in interpreting the biological effects observed in animal models.

Liquid chromatography with UV detection is a conventional method to separate and quantify vitexin and isovitexin from leaf extracts [13,14]. HPLC-UV was also used to measure the concentration of vitexin or isovitexin separately in plasma and tissue after its intravenous administration on rats by using protein precipitation, evaporation, and reconstitution [13,15]. Later, liquid chromatography tandem mass spectrometry was established for the simultaneous determination of vitexin and isovitexin in rat plasma after oral administration of *Santalum album* L. leaf extract [3]. Similar pharmacokinetic studies on vitexin and isovitexin have been conducted on rats [16,17,18,19]. The pharmacokinetic assessment of new improved-absorption formulations (e.g., encapsulation) in rabbits is more practically feasible and provides reliable pharmacokinetics results that can be replicated [20]. However, these previous studies have some limitations. Their procedures are time-consuming and require large injection volumes onto the chromatographic column to achieve the necessary sensitivity.

Therefore, this study aims to develop and validate a sensitive bioanalytical method that minimizes the amount of sample matrix injected onto the column. The method is specifically designed to evaluate the pharmacokinetics of vitexin and isovitexin in rabbits, facilitating the investigation of new formulations and advancing their potential therapeutic applications.

## 2. Results

### 2.1. LC-ESI-MS/MS Method Development

**Figure 2:**
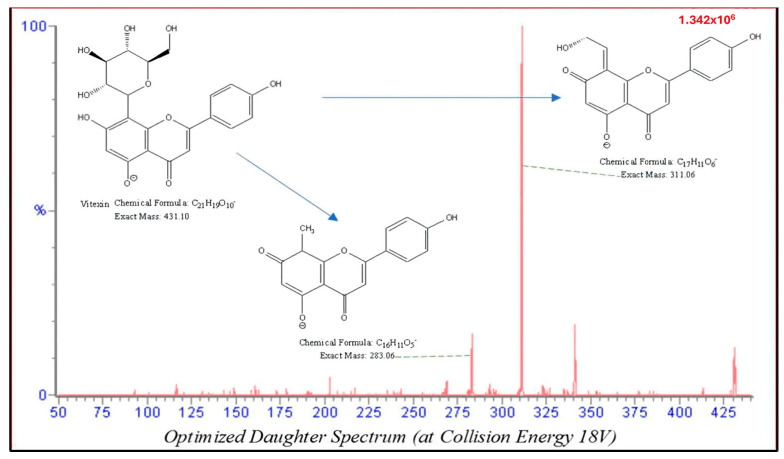
Daughter spectrum and proposed fragmentation mechanism of Vitexin (Collision energy 18 V).

A mixture of standards and internal standard at a concentration of 1 µg/mL was ana-lyzed in full-scan mode to identify the parent ions, which are deprotonated molecule ions [M − H]^−^ losing a C_4_H_8_O_4_ group, at *m*/*z* 431 for both analytes under the MS/MS scan mode. The relatively weak bonds in the glucose structure make their molecules prone to break out in the collision cell, resulting in the formation of fragmented ions. Daughter ion scan mode was used to determine both quantitative and qualitative fragment ions. The most abundant product ions of isovitexin and vitexin were the result of [M–H–C_4_H_8_O_4_]– and [M–H–C_4_H_8_O_4_]^−^, respectively, at *m*/*z* both 311.1 (Table 1). Thus, the MRM transitions were chosen at 431.1 → 311.1 for monitoring the compounds. Fragmentations of the parent ions into daughter ions were consistent with data found in MassBank of North America (MoNA) and our proposed fragmentation mechanism (Figure 2). Fragmentation parameters and collision energy were optimized using the IntelliStart automatic optimization function of MassLynx (V4.2) software and further adjusted with Manual Tune to maximize the signal intensity of the quantitative ions.

### 2.2. Method Validation

#### 2.2.1. System Suitability

To assess system suitability, an MQC sample was injected six times. The results showed that the relative standard deviation (RSD%) of retention times, retention time ratios, and peak area ratios of the analytes and internal standard were all below 5.0% after six consecutive analyses. Detailed results are presented in Table 2. Therefore, the LC-MS/MS system meets the suitability criteria for analysis.

#### 2.2.2. Specificity and Selectivity

**Figure 3:**
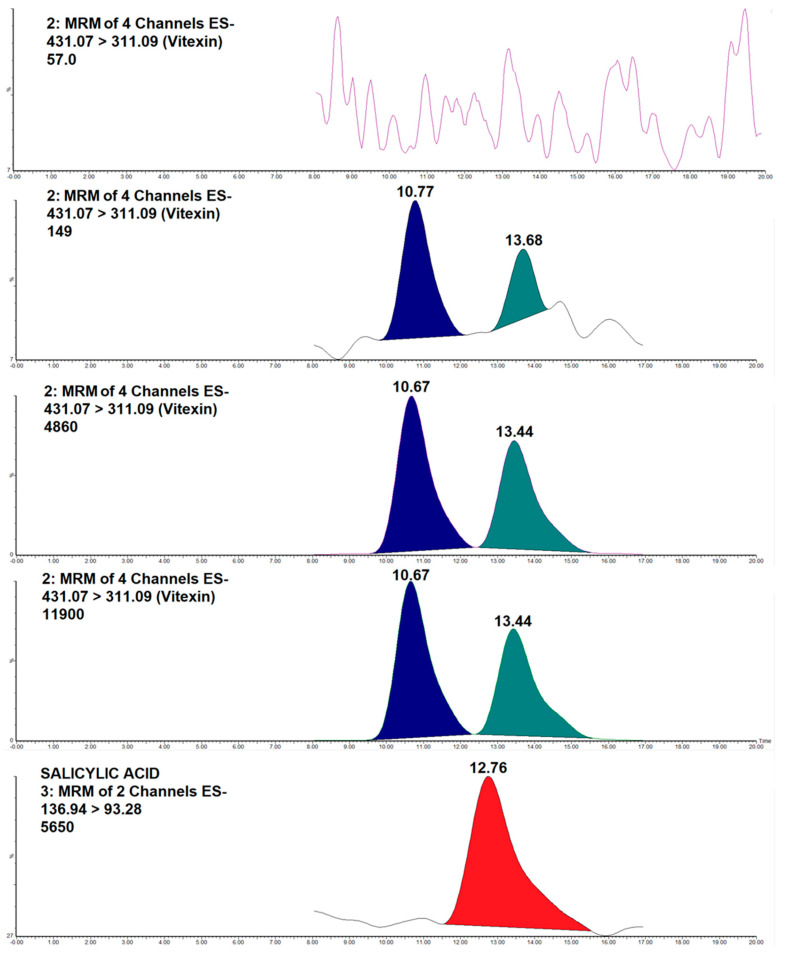
Chromatograms obtained for the transition 431 > 311 (vitexin and isovitexin) from the blank, LLOQ, MQC, and ULOQ level, and for the transition 137 > 93 (IS) at the LLOQ level.

Both analytes had the same transition ions, including parent ions and daughter ions (quantitative and qualitative ions). Therefore, it is crucial to separate the analytes on chromatograms. At the LLOQ concentration level, the chromatogram of our samples showed a vitexin peak at Rt = 10.67, while an isovitexin peak was recorded at Rt = 13.44. All the peaks were completely separated via the chromatogram. Chromatograms of blank plasma showed no peak at the positions of our analytes and IS. Figure 3 provides chromatograms of blank plasma sample and control plasma samples as follows: LLOQ (t_1_ = 10.77, t_2_ = 13.68, w_1_ = 1.04, w_2_ = 0.70, R_S_ = 1.97), MQC (t_1_ = 10.67, t_2_ = 13.44, w_1_ = 1.04, w_2_ = 1.0, R_S_ = 1.60), and ULOQ (t_1_ = 10.77, t_2_ = 13.44, w_1_ = 1.09, w_2_ = 1.20, R_S_ = 1.43).

#### 2.2.3. Linearity

Calibration curves were designed to cover the ranges of 2.0–200.0 ng/mL. The calculation shows a strong correlation (R^2^ > 0.99) between the response and the concentration of the analytes, with a weighting factor of 1/x^2^. The calibration equation utilized in this study, which incorporates an internal standard (IS), follows the form y = ax + b. The specific concentration ranges and individual equations for each analyte can be found in Table 3.

#### 2.2.4. Accuracy and Precision

The intra- and inter-day precision data summarized in Table 4 suggest that our results were reliable. The LLOQ was selected based on Tmax to demonstrate the superior efficiency of the microcapsule formulation. At a concentration of 2.0 ng/mL for both analytes in plasma, the results demonstrated good accuracy and precision. Therefore, this concentration was identified as the lower limit of quantification (LLOQ) for the method.

#### 2.2.5. Extraction Efficiency and Matrix Effect

During protein precipitation, proteins were effectively eliminated from the samples while the analytes and IS remained intact, resulting in absolute recovery rates of 97–102%. Despite the presence of various polar impurities, MF values maintained at ideal levels (90–100%). The IS-normalized MF across six different lots of samples demonstrated excellent stability, with RSDs of less than 7% (Table 4).

#### 2.2.6. Carry-Over

**Figure 4:**
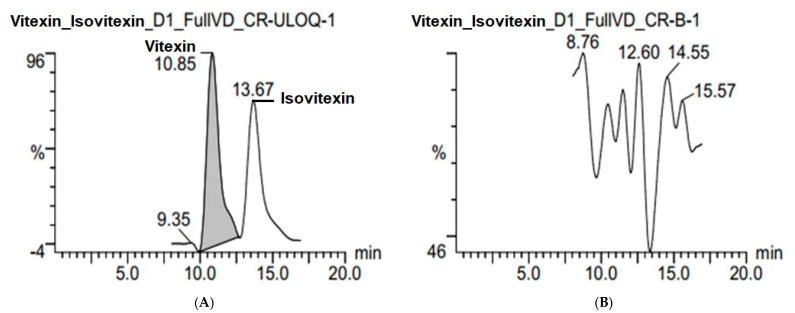
Chromatograms of blank sample (**B**) after injecting ULOQ sample (**A**).

The chromatogram of any blank sample analyzed immediately after the injection of the ULOQ sample shows no peak signals at the retention times of any analytes or the internal standard (Figure 4). This indicates that carry-over does not affect the analytical results.

#### 2.2.7. Dilution Integrity

After dilution from a concentration of 2× HQC with blank rabbit plasma, the accuracy remained within the range of 92–95%, with an RSD of less than 5%, indicating that a dilution factor of 2 does not compromise the analytical results (Table 4).

#### 2.2.8. Stability

The stability of the analytes was extensively evaluated in plasma samples and wet extract samples under various storage conditions to simulate real-world storage scenarios. Vitexin and isovitexin were encountered to be stable in rabbit plasma for a long time in normal and harsh conditions, particularly for up to 8 h at room temperature, for 24 h in autosampler condition after sample preparation, for up to 1 month in frozen temperature (<−20 °C), and for three freeze-thaw cycles (Table 5).

### 2.3. Application of the Method for Pilot Testing of Biological Samples

**Figure 5:**
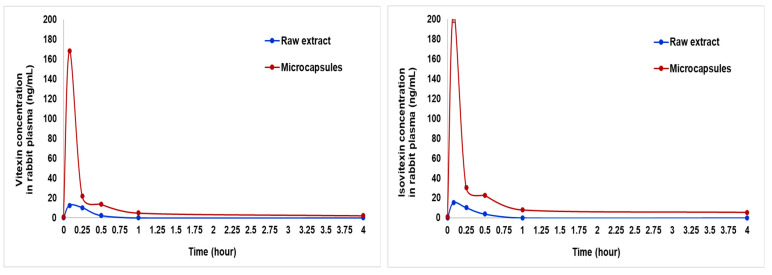
Pharmacokinetic curves of vitexin and isovitexin in rabbits after oral administration of raw extract and microcapsules at a dose of 15 mg/kg. Data were presented as Mean ± S.E.M. Data are expressed as mean ± S.E.M. and analyzed using *t*-test (n = 2 per group).

The developed method was applied to analyze biological samples extracted from rabbit plasma as a part of a simulated pharmacokinetic study on a pilot scale. As shown in Figure 5, the encapsulated form of vitexin exhibited a similar Tmax but achieved a nearly 8-fold higher Cmax compared to the raw form. Likewise, the encapsulated form of isovitexin reached a Cmax that was 10 times higher at a similar Tmax to its raw counterpart. Additionally, the time to reach Cmax for vitexin and isovitexin from the encapsulated form was faster than that observed for their extract forms [21].

## 3. Discussion

Three typical extraction methods, including protein precipitation (PP), liquid-liquid extraction (LLE), and solid-phase extraction (SPE), can be used to tract the compounds from the raw rabbit plasma. SPE was not selected due to economic considerations. Both analytes are polar flavonoid glycosides (estimated logP ≈ 0.1), and their extraction from the polar plasma matrix using organic solvents (LLE) is highly inefficient. PP was proved to be the most suitable for extracting vitexin and isovitexin from plasma. In this method, raw rabbit plasma samples were deproteinized with acetonitrile as the precipitation agent to remove proteins, which could increase the extraction efficiency (97–102%). Additionally, PP provided sufficient sensitivity for our analysis. Therefore, PP was chosen as the sample preparation method for this study.

Both vitexin and isovitexin are polar flavonoid-glycoside compounds, and belong to the C-glycosylflavones. Although they share the same molecular formula, their structures differ in the position of the sugar group. Due to this structural similarity, both analytes produce the same parent ion when detected using a TripleQuad mass spectrometer with an ESI source (Waters, Milford, MA, USA). Moreover, it was observed that both compounds also generate the same fragmented ions, resulting in the same signal on the MS detector across transitions. Therefore, it is crucial to achieve separation of vitexin and isovitexin with liquid chromatography before detection to ensure accurate identification and quantification of each analyte. Theoretically, both vitexin and isovitexin are polyphenol compounds and more readily ionized in negative mode compared to positive mode. In practice, their ion intensity was indeed high in negative mode in an ESI full scan; therefore, the negative mode with an ESI source was selected as the ionization mode for this study to optimize detection sensitivity and reliability. This mode was also chosen for analyzing these compounds in the plant materials [2] as well as rat plasma [3] in previous studies.

Due to ion flow variability in mass spectrometry detectors, using an internal standard is essential to enhance accuracy, precision, and control the analytical process. Internal standards are categorized into two types: isotopic and analog. Isotopic internal standards are structurally identical to the analyte but contain isotopic labels. While isotopic standards are theoretically optimal, they may not be available for newly analytes. In such cases, an analog internal standard with similar properties to the analytes, regarding extraction (solubility), chromatographic analysis (retention time), and MS detection (ionization mode), was chosen. Based on the chemical formula, log distribution coefficient, and pKa values of various compound, salicylic acid was selected as the internal standard for the method.

Our developed analytical approach demonstrated high sensitivity in order to effectively evaluate, simultaneously, concentrations of vitexin and isovitexin in advanced drug delivery systems. Previous analytical procedures have some limitations when applied to biological samples such as plasma [3,17,18,19]. In their sample preparation, the evaporation step is time-consuming due to the presence of water and protein-precipitation solvents (methanol and acetonitrile), which are difficult to evaporate efficiently. Additionally, their methods required large injection volumes (greater than 10 µL) onto the chromatographic column to achieve the necessary sensitivity. This can adversely affect column durability, potentially reducing its lifespan and overall performance. Finally, our findings reveal the insignificant matrix effect on the ionization of both vitexin and iso-vitexin. Compared to a study on rat plasma using puerarin as the internal standard [3], our procedure gives higher recoveries, even in very small concentrations of vitexin and isovitexin (2–6 ng/mL) that could not be detected in the rat plasma.

This pilot study obtains highly reliable results, which means our procedure is versatile to simultaneously quantify vitexin and isovitexin at very low concentrations in plasma samples from large animals. Vitexin and isovitexin are natural flavonoids with promising biological activities in vitro, but the poor gastrointestinal absorption and low oral bioavailability significantly limit their potential therapeutic applications. The results show that our encapsulated extracts significantly enhanced the Cmax of vitexin and isovitexin in rabbits. This suggests that the microcapsule delivery system can significantly improve dissolution and bioavailability, addressing the challenges of poor absorption and low oral bioavailability associated with vitexin due to its rapid clearance from the bloodstream. This is regarded as a successful pilot study for further in vivo studies with larger sample sizes and large animal models to ensure greater reliability when applying this analytical procedure in different pharmacokinetic models.

## 4. Materials and Methods

### 4.1. Chemicals and Materials

Vitexin and isovitexin were isolated from Mung bean seed coat with a purity of greater than 95%. Vitexin standards and salicylic acid as internal standard (IS) were obtained from the Institute of Drug Quality Control in Ho Chi Minh City (Vietnam). All the solvents for the mobile phases, extraction, and sample diluents were of high purity and suitable for HPLC analyses, supplied by J.T. Baker (Avantor, NJ, USA). Other chemicals include alginate (TCI, Tokyo, Japan), chitosan (Sigma-Aldrich, Steinheim, Germany), isooctane, Tween 80, Span 80, calcium chloride, acetone, and acetic acid (Fisher, Dreieich, Germany).

### 4.2. Preparation of Chitosan-Coated Alginate Microcapsules

The extracted and HPLC-purified vitexin and isovitexin were encapsulated in the ratio of 1:1 with the following process. Alginate cores were synthesized using the water-in-oil (W/O) emulsion technique [22]. Then, 200 mg of vitexin-isovitexin mixture was dispersed and thoroughly mixed into the alginate solution. This suspension was carefully dripped into isooctane (40.00 g) containing Span 80 (2.11 g) on a homogenizer T25—Digital Ultra-Turrax (IKA, Staufen, Germany) at 7200 rpm to create a W/O emulsion. Tween 80 solution (5.78 g) and CaCl_2_ 7.6% were added, respectively, while the mixture was kept homogeneous. This triggered the gelation of the alginate to form the alginate microcapsule cores. Impurities were removed by solidification in acetone, centrifuging, and filtration. The cores were washed with acetone, dried at room temperature, and subsequently immersed in chitosan solution (0.5% *w*/*w* in acetic acid 1.0% *w*/*w*, pH 5) with a 1:100 ratio. Magnetic stirring at approximately 1000 rpm was applied for 2 h at room temperature to facilitate the coating process. The coated microcapsules were then washed repeatedly with distilled water to eliminate any residual chitosan on the surface and subsequently dried at 37 °C. Finally, the chitosan-coated alginate microcapsules were stored in a sealed bottle under dry conditions for further analysis and experimentation.

### 4.3. Instrumentation and Chromatographic Conditions

The sample analysis was performed on an HPLC Wates Acquity Class I Plus system (Waters, Milford, MA, USA) equipped with a binary pump, an autosampler, and a temperature-controlled column compartment. The sample was kept at 10 °C in an autosampler until analyzed. The liquid chromatographic separation was performed on a Waters (Milford, MA, USA) XTERRA RP18 column (100 mm × 4.6 mm, 3.5 µm), employing a mixed mobile phase comprising methanol-0.1% acetic acid in the ratio of 40:60 (*v*/*v*) with an isocratic elution of 0.35 mL/min and a system pressure of 2200 psi. The injection volume was 2.0 µL. Detection was carried out with a triple quadrupole mass spectrometer Xevo TQ-S micro (Waters, Milford, MA, USA) equipped with an electrospray ionization (ESI) source and operated in negative mode [3].

The following optimized source parameters were set: capillary voltage at 2.0 kV, desolvation gas flow at 1000 L/h, desolvation temperature at 500 °C, and cone gas flow at 150 L/h. Quantification was monitored with multiple reactions monitoring (MRM). Both vitexin and isovitexin produced parent ions at *m*/*z* 431, while salicylic acid (the internal standard) produced a parent ion at *m*/*z* 137. These parent ions were fragmented in the collision cell to yield product ions at *m*/*z* 311 for vitexin and isovitexin, and at *m*/*z* 93 for salicylic acid. MassLynx version V4.2 was used to control all parameters of LC and MS/MS.

### 4.4. Plasma Sample Preparation

Before extraction, the frozen samples were thawed to room temperature. To 200 µL of the samples, 200 µL of IS solution 1 µg/mL in acetonitrile was added to cause protein precipitation. Thereafter, the mixture was vortexed for 30 s and centrifuged at 15,000 rpm for 5 min. The supernatant was filtered through nylon filters with a pore size of 0.22 µm into vials and kept at 10 °C in an autosampler until analyzed.

### 4.5. Quality Control (QC) Samples

Quality control samples were prepared by spiking standard solutions to plasma. Simulated samples were prepared with a dilution of 1/20. Calibration standards of vitexin and isovitexin from 2.0 to 200.0 ng/mL were prepared in methanol. QC samples were prepared at four concentration levels: 2.0 ng/mL (lower limit of quantification, LLOQ), 6.0 ng/mL (low quality control, LQC), 80.0 ng/mL (medium quality control, MQC), and 160.0 ng/mL (high quality control, HQC) for both analytes.

### 4.6. Method Validation

The method was validated in accordance with the FDA requirements, including specificity, selectivity, linearity, recovery, accuracy, precision, low limit of qualification, carry-over, stability [23], and matrix effect according to EMA guidelines [24].

#### 4.6.1. System Suitability

A processed MQC sample was injected six times consecutively. The relative standard deviation (RSD%) of the retention time ratio (analytes to IS) and the peak area ratio (analytes to IS) were calculated. The deviation should not exceed 5%.

#### 4.6.2. Specificity, Selectivity

Six blank rabbit plasma and six LLOQ samples from each batch were analyzed. Signals from endogenous substances in the blank samples were considered acceptable if not exceeding 20.0% of the signal observed in the LLOQ samples. MassLynx (V4.2) software does not provide the resolution; it was calculated by the formula:

$$R_{S}=1.18\times (\frac{t_{R_{2}}-t_{R_{1}}}{W_{{0.5h}_{1}}+W_{{0.5h}_{2}}})$$

#### 4.6.3. Linearity

In each run, a set of eight calibration standards in the range of 2.0–200.0 ng/mL was analyzed. A linear equation was established by plotting the area ratio (analyte to IS) against the nominal analyte concentration. The correlation coefficient (r) must be greater than 0.99 (or r^2^ > 0.9801). The acceptable range for deviation concentrations should be within +15%, and the LLQQ should be between ±20% [25,26].

#### 4.6.4. Accuracy and Precision

Each run was performed on LLOQ, LQC, MQC, and HQC in six replicates. Accuracy was evaluated by intraday and interday assessments on the recovery of the measured analyte concentration to its nominal value. Acceptable accuracy should be within the range of 85.0–115.0%, with the exception of the LLOQ (80.0–120.0%). Precision was determined by calculating the RSD of the measured concentrations (or recoveries) at each level. The RSDs at all concentrations could not exceed 15.0%, except for LLOQ (could not exceed 20%) LLOQ was determined based on accuracy and precision at the LLOQ concentration level. Additionally, the signal-to-noise (S/N) ratio of analytes must be greater than 5.0.

#### 4.6.5. Extraction Efficiency and Matrix Effect

Three types of samples were tested at LQC and HQC levels: simulated samples, spiked post-extraction samples from six lots of rabbit plasma, and spiked solvent samples. The following formula was used to obtain each lot’s matrix factor for the analyte and IS:

Extraction efficiency (RE) = Ssimulated/Sspiked × 100 (%)

Matrix factor (MF) = Sspiked/Ssolvent × 100 (%)

An ideal procedure should have both RE and matrix factor MF values close to 100%. To evaluate the matrix effect, the IS-normalized MF was calculated by dividing the analyte’s MF by the IS’s MF. Extraction efficiency and matrix effect were calculated from six different plasma lots; RSD% should remain below 15%.

#### 4.6.6. Carry-Over

A sample at the upper limit of quantification (ULOQ) and a blank sample were injected, each in six replicates. At the retention time of each analyte, the peak reaction of the blank sample should be less than 20% of the analyte’s peak response, and less than 5% of the IS response in the LLOQ sample.

#### 4.6.7. Dilution Integrity

Six samples at a concentration of 2× HQC were diluted two-fold with rabbit blank plasma and analyzed using the developed method. The results must meet the specified requirements for accuracy and precision.

#### 4.6.8. Stability

The stability of the analytes was thoroughly evaluated in rabbit plasma samples under various storage conditions, including bench-top (20–30 °C, 8 h), three freeze-thaw cycles, and long-term storage at below −20 °C for 30 days. Additionally, processed samples were stored in the autosampler at 10 °C to assess stability during the extraction process. At each storage condition, samples at LQC and HQC were tested in six replicates. All levels must meet the specified requirements for accuracy and precision.

### 4.7. Applications in Pharmacokinetic Study

Male New Zealand rabbits weighing 2.5 ± 0.5 kg with no abnormalities or malformations were purchased from Thuan Giang Trading Service Company (Ho Chi Minh City, Vietnam). After transportation to the housing facility for experiment preparation, the rabbits were acclimatized for 7 days in individual metallic wire cages with food and water provided ad libitum at the Department of Pharmacology, Faculty of Pharmacy, University of Medicine and Pharmacy at Ho Chi Minh City. During this period, environmental conditions such as temperature, humidity, and lighting were maintained consistently, and the rabbits were regularly familiarized to minimize stress. Prior to the experiment, the rabbits were fasted for 12 h to minimize food-related effects on drug absorption. They were fed 4 h after drug administration to ensure that food intake did not interfere with the drug absorption process. The rabbits were kept in air-conditioned metal cages (70 × 50 × 40 cm cage, temperature 25–30°C) and had free access to water and food provided by Pasteur Institute of Ho Chi Minh City for a week prior to the experimentation at the Department of Pharmacology, Faculty of Pharmacy, University of Medicine and Pharmacy at Ho Chi Minh City.

The first group was intragastrically administered microencapsulated vitexin-isovitexin 15 mg/kg body weight, while the second one received regular vitexin-isovitexin with the same dose and method [10]. Some 1 mL of blood samples was collected from the marginal ear vein using a 20 g intravenous catheter at the time points 0 min, 5 min, 15 min, 30 min, and 1 h, 2 h, 4 h after treatment [7,8,9], and transferred into tubes containing anticoagulant (EDTA). The samples were centrifuged at 3000 rpm for 15 min to harvest rabbit plasma, which was then transferred into clean Eppendorf tubes and stored at −20 °C until analysis [3,10].

## 5. Conclusions

The LC-MS/MS method established in this study offers a reliable, sensitive, and accurate tool for simultaneously measuring the concentrations of two C-glycoside flavonoids in rabbit plasma for the first time. This versatile quantification of levels of vitexin and iso-vitexin in biological samples is essential to evaluate the efficacy of new, im-proved absorption formulations after oral administration. This advancement paves the way for achieving enhanced therapeutic outcomes for these promising natural agents.

## Figures and Tables

**Table 1:** Critical tandem mass spectrometer parameters.

Compound	Parent Ions (*m*/*z*)	Cone (V)	CE (V)	Daughter Ions (*m*/*z*)	
				Quantitative Ions	Qualitative Ions
Vitexin	431	40	18	311	283
Isovitexin	431	40	18	311	283
Salicylic acid (IS)	137	6	20	93	-

**Table 2:** Results of system suitability test using six repeated MQC injections.

No.	Vitexin			Isovitexin		
	Retention Time (min)	Ratio (Analyte/IS)		Retention Time (min)	Ratio (Analyte/IS)	
		Retention Time	Area		Retention Time	Area
1	10.71	0.823	0.934	13.48	1.036	0.743
2	10.71	0.821	0.872	13.53	1.037	0.709
3	10.76	0.825	0.899	13.53	1.037	0.718
4	10.76	0.818	0.876	13.53	1.029	0.701
5	10.76	0.818	0.880	13.57	1.032	0.710
6	10.76	0.821	0.868	13.57	1.036	0.697
Mean	10.74	0.821	0.888	13.54	1.034	0.713
SD	0.03	0.00	0.02	0.03	0.00	0.02
CV (%)	0.24	0.31	2.79	0.25	0.31	2.30

**Table 3:** Linearity for analytes in rabbit plasma.

	Range (ng/mL)	Equation y = ax + b, Weighting Factor 1/x^2^		
		a	B	R^2^
Vitexin	2.0–200.0	0.0114	−0.0027	0.9928
Isovitexin	2.0–200.0	0.0092	−0.0043	0.9961

**Table 4:** Results of validation for recovery, matrix effect, accuracy, and precision.

Compound	Theoretical Concentration (ng/mL)	Accuracy (%)		Recovery (%) (n = 6)	Matrix Effect (n = 6)	
		Intraday (n = 6)	Interday (3 days, n = 18)		Matrix Factor (MF) (%)	IS-Normalized MF
Vitexin	2.0	110.15 (3.22)	106.36 (7.66)			
	6.0	104.97 (6.29)	103.28 (1.43)	99.12 (10.72)	100.06 (6.67)	102.93 (5.71)
	80.0	96.41 (3.2)	95.06 (1.97)	98.13 (2.15)		
	160.0	101.98 (3.83)	103.09 (3.22)	97.53 (1.49)	90.86 (1.52)	93.48 (6.88)
	320.0 (diluted × 2)	92.38 (4.37)	-	-	-	-
Isovitexin	2.0	104.06 (8.7)	101.52 (7.16)	-	-	-
	6.1	104.11 (5.28)	102.35 (1.82)	101.98 (6.75)	90.38 (6.94)	93.00 (6.53)
	81.0	94.32 (2.41)	94.5 (0.72)	99.37 (2.29)	-	-
	161.9	100.59 (3.44)	102.98 (2.33)	99.74 (1.59)	97.74 (1.52)	100.55 (6.88)
	323.8 (diluted × 2)	94.19 (3.87)	-	-	-	-
Salicylic acid (IS)		-	-	97.07 (2.04)	97.46 (6.44)	-

Data presented as mean (RSD).

**Table 5:** Stability of analytes and IS in plasma and wet extraction.

Compound	Conc. (ng/mL)	Plasma			Wet Extract
		Bench-Top	Freeze-Thaw	Long-Term	Autosampler
		(20–30 °C, 8 h)	(3 Cycles)	(<−20 °C, 30 Days)	(10 °C, 24 h)
Vitexin	6.0	104.52 (7.26)	96.46 (7.22)	91.94 (2.13)	102.34 (5.6)
	160.0	107.2 (5.24)	103.92 (2.94)	92.60 (2.39)	101.02 (3.58)
Isovitexin	6.1	100.7 (6.56)	94.48 (6.36)	95.00 (7.36)	102.57 (8.64)
	161.9	106.8 (5.6)	106.38 (2.59)	94.92 (2.43)	101.02 (3.5)
IS	1000	108.16 (3.17)			95.31 (3.72)

Data presented as mean (RSD).

## Data Availability

The data presented in this study are available upon request from the corresponding author.

## Abbreviations

The following abbreviations are used in this manuscript:

AUC	Area under curve
CE	Collision energy
EMA	European medicines agency
ESI	Electrospray ionization
FDA	(Us) food and drug administration
HPLC	High performance liquid chromatography
HQC	High quality control
LC	Liquid chromatography
LLOQ	Lower limit of quantification
LQC	Low quality control
MQC	Middle quality control
MS	Mass spectrometry
RSD	Relative standard deviation
ULOQ	Upper limit of quantification
UV	Ultraviolet
